# Characteristics and survival of patients with advanced cancer and p53 mutations

**DOI:** 10.18632/oncotarget.2004

**Published:** 2014-05-25

**Authors:** Rabih Said, Yang Ye, David S. Hong, Filip Janku, Siqing Fu, Aung Naing, Jennifer J. Wheler, Razelle Kurzrock, Christoforos Thomas, Gary A. Palmer, Kenneth R. Hess, Kenneth Aldape, Apostolia M. Tsimberidou

**Affiliations:** ^1^ Department of Investigational Cancer Therapeutics (Phase I Clinical Trials Program), The University of Texas MD Anderson Cancer Center, Houston, TX; ^2^ Department of Internal Medicine, Oncology Division, The University of Texas Health Sciences Center, Houston, TX; ^3^ Center for Personalized Therapy and Clinical Trials, University of California San Diego - Moores Cancer Center, San Diego, CA; ^4^ Department of Biology and Biochemistry, University of Houston, Houston, Texas; ^5^ Foundation Medicine, Cambridge, MA; ^6^ Department of Biostatistics, The University of Texas MD Anderson Cancer Center, Houston, TX; ^7^ Department of Pathology, The University of Texas MD Anderson Cancer Center, Houston, TX

**Keywords:** p53 mutations, matched therapy, molecular aberrations

## Abstract

P53 mutations are associated with invasive tumors in mouse models. We assessed the p53mutations and survival in patients with advanced cancer treated in the Phase I Program. Of 691 tested patients, 273 (39.5%) had p53 mutations. Patients with p53 mutations were older (p<.0001) and had higher numbers of liver metastases (p=.005). P53 mutations were associated with higher numbers of other aberrations; PTEN (p=.0005) and *HER2 (*p=.003)aberrations were more common in the p53 mutation group. No survival difference was observed between patients with p53 mutations and those with wild-type p53. In patients with wild-type p53 and other aberrations, patients treated with matched-therapy against the additional aberrations had longer survival compared to those treated with non-matched-therapy or those who received no therapy (median survival, 26.0 vs. 11.8 vs. 9.8 months, respectively; p= .0007). Results were confirmed in a multivariate analysis (p= .0002). In the p53 mutation group with additional aberrations, those who received matched-therapy against the additional aberrations had survival similar to those treated with non-matched-therapy or those who received no therapy (p=.15). In conclusion, our results demonstrated resistance to matched-targeted therapy to the other aberrations in patients with p53 mutations and emphasize the need to overcome this resistance.

## INTRODUCTION

The tumor suppressor protein p53 is activated in response to a variety of stress signals to ensure genome stability in cells. The role of p53 in guarding the cell reflects its ability to act as a potent transcriptional activator, regulating the expression of genes that inhibit cell cycle progression or induce apoptosis or senescence. By eliciting cell growth inhibition, p53 functions to prevent cancer development [1]. In addition to its role in suppressing tumor growth, p53 can limit cancer progression by decreasing the invasiveness and metastatic capacity of cancer cells [2-4].

Loss of p53 function occurs frequently in human cancers and results from mutations in the p53 gene or defects in the pathway that activates p53 [1]. More than 75% of p53 mutations lead to the expression of a full-length protein with a single amino acid substitution. In most cases, these mutant p53 proteins lose wild-type functions, elicit dominant-negative effects on the remaining wild-type p53, or acquire oncogenic functions [5-7]. This gain-of-function phenotype has been characterized in *in vivo* studies. Mice that carry mutant p53 develop different types of tumors with more invasive phenotypes than the *p53*-null mice in which the p53 protein is not expressed [6, 8]. In addition, the analysis of cell culture models has identified several oncogenic properties of mutant p53, including its ability to promote migration, invasion, angiogenesis, and chemoresistance [2-4, 7, 9, 10]. The single amino acid substitutions in the p53 protein are divided into two broad classes: structural and contact DNA mutations. Most of the point mutations, including those at amino acids R175, G245, R248, R249, R273 and R282, tend to cluster at hot spots within the DNA-binding domain. Contact hot spot mutations lead to the loss of DNA binding activity and subsequent alterations in the transcriptional activity of p53. Structural hot spot mutations cause unfolding of the p53 protein that affects its interaction with other proteins. Mutant p53 has been shown to elicit its oncogenic actions by interacting with and inhibiting its family members, p63 and p73. Mutant p53 is known to alter the expression of p63 target genes, and this has been associated with enhanced invasive behavior [7]. In addition, oncogenic mutations that activate the Ras signaling pathway inhibit the function of p63 by promoting its interaction with mutant p53. By regulating the activity of mutant p53, oncogenic Ras induces the development of prometastatic phenotypes[11, 12].

Several studies have investigated the importance of p53 mutation status in predicting clinical outcome in various types of cancer. Some of the p53 mutations have been correlated with shorter survival or a poorer response to treatment in several cancers[13]. In addition, some mutations were correlated with worse survival than other mutations. For example, mutations in the DNA-binding domain of p53 have been associated with worse prognosis and poorer response to chemotherapy [14].

We have previously reported the clinical characteristics and response to standard systemic therapy of 145 patients with documented p53 mutational status (n=66 mutated vs. n=79 wild-type tumors) who were referred to our department for participation in Phase I clinical trials[15]. In the current study, we assessed the frequency of various p53 mutations and their effects on clinical outcomes in a larger number of patients with advanced cancer who were referred for treatment to the Phase I Clinical Trials Program at The University of Texas MD Anderson Cancer Center. We also evaluated the impact on clinical outcomes of additional targetable aberrations in patients with and without p53 mutations.

## RESULTS

### Demographics

From May 2010 to April 2013, 691 patients were found to have tumor tissue tested for p53 mutation status. Of these, 273 (39.5%) had p53 mutations and 418 (60.5%) had wild-type p53. The patients' baseline characteristics by p53 mutation status are shown in Table 1a and Table 1b. Patients with p53 mutations were older (median age, 59 vs. 55 years, p<.0001) and were more likely to have liver metastases (108/273 [33%] vs. 122/418 [29%], p=.005) compared to patients with wild-type p53.

**Table 1a:** Characteristics of patients with wild-type and mutant p53 tumors

	P53 mutation N=273 (%)	P53 wild-type N=418 (%)	Total N=691 (%)	p-value
Sex				.2
Female	141 (52)	236 (56)	377 (55)	
Male	132 (48)	182 (44)	314 (45)	
Age				.01
>60	130 (48)	159 (38)		
Race				.6
White	207 (76)	318 (76)	525 (76)	
Hispanic	29 (11)	37 (9)	66 (10)	
Black	27 (10)	39 (9)	66 (10)	
Asian	8 (2)	18 (4)	26 [16]	
Unknown	2 (1)	6 (2)	8 (1)	
Tumor type				NA
CRC	49 (18)	52 (12)	101 (15)	
Ovarian	31 (11)	27 (7)	58 (8)	
GI, other	29 (11)	28 (7)	57 (8)	
Breast	22 (8)	43 (10)	65 (9)	
GYN, other	18 (7)	17 (4)	35 [16]	
Lung	18 (7)	22 [16]	40 (6)	
Genitourinary	17 (6)	29 (8)	46 (7)	
Head and neck	15 [16]	53 (12)	68 (10)	
Sarcoma	9 [16]	45 (11)	54 (8)	
Endometrial	9 [16]	12 [16]	21 [16]	
Melanoma	8 [16]	22 [16]	30 (4)	
Pancreatic	5 (2)	9 (2)	14 (2)	
Thyroid	4 (1)	9 (2)	13 (2)	
Other	39 (14)	50 (12)	89 (13)	
ECOG score				.23
0	69 (25)	128 (31)	197 (29)	
1	180 (66)	247 (59)	427 (62)	
2	19 (7)	35 (8)	54 (8)	
3	3 (1)	1 (−)	4 (1)	
Unavailable	2 (1)	7 (2)	9 (1)

**Table 1b:** Characteristics of patients with wild-type and mutant p53 tumors

Albumin				.55
≥ 3.5 g/dL	238 (87)	369 (88)	607 (87)	
<3.5 g/dL	26 (10)	47 (11)	73 (10)	
Unavailable	9 [16]	4 (1)	23 [16]	
LDH				.35
>618 U/L	90 (33)	128 (31)	218	
≤618 U/L	172 (63)	286 (68)	458	
Unavailable	11 (4)	4 (14)	27	
RMH score				.59
0	126 (46)	212 (51)	338 (49)	
1	87 (32)	123 (29)	210 (30)	
2	42 (16)	69 (17)	111 (16)	
3	6 (2)	9 (2)	15 (2)	
Unknown	12 (4)	5 (1)	17 [16]	
Liver metastases	108 (40)	122 (29)	230 (33)	.005
Number of metastatic sites				
Median (Range)	2 (0-8)	2 (0-7)	2 (0-8)	.15
Number of prior therapies				
Median (Range)	3 (0-10)	3 (0-13)	3 (0-10)	.16

Abbreviations: CRC-colorectal, GI-gastrointestinal, GYN-gynecological, ECOG-Eastern Cooperative Oncology Group, LDH-lactate dehydrogenase, RMH-Royal Marsden Hospital.

No difference between the two groups was observed in sex, race, performance status, number of metastatic sites, number of prior therapies, serum albumin and lactate dehydrogenase levels, and Royal Marsden Hospital score. The most common tumor types seen were colorectal, head and neck, and breast cancers, which reflected the referral pattern of patients in the Phase I Clinic. Colorectal cancer was the most common malignancy in both groups (wild-type, n=49 [18%] and mutant, n=52 [12.5%]).

### Distribution of p53 Mutations

**Figure 1:**
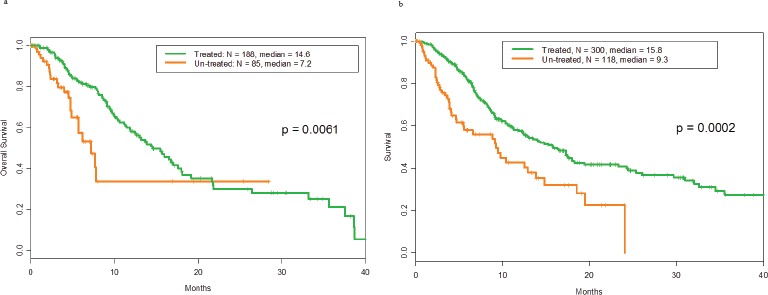
**a** Overall survival of patients with mutant p53 tumorsby whether they did or did not receive any treatment in phase I Of 188 patients treated with phase 1 trials the median overall survival was 14.6 months. Of 85 patients non-treated the median overall survival was 7.2 months.b. Overall survival of patients with wild-type p53 tumors by whether they did or did not receive any treatment in phase I. Of 300 patients treated with phase 1 trials the median overall survival was 15.8 months. Of 118 patients non-treated the median overall survival was 9.3 months.

Of 273 patients with p53 mutations, 17 (6%) had more than one p53 mutation. The distribution of p53 mutations by exons, codons, and tumor types is summarized in Table 2. Of the 273 patients, 239 (84%) had mutations within the DNA-binding domain, with the following distribution: exon 5 (n=73, 27%), exon 6 (n=45, 16%), exon 7 (n=63, 23%), and exon 8 (n=58, 21%). Mutations within other exons were found in 47 (17%) patients. P53 mutations within exon 5 were more common in colorectal cancer, whereas mutations within exons 6, 7, and 8 were more common in lung, pancreatic, and ovarian cancer, respectively. Mutations within other exons were more common in various gynecological, breast, and genitourinary tumors (Supplemental - Figure 1e).

**Table 2:** Distribution of p53 mutations by exons, codons, and tumor types

	Exon Location*					Codon Location*		
Cancer type	Exon 5 (%)	Exon 6 (%)	Exon 7 (%)	Exon 8 (%)	Others (%)	Hot-spot (%)	DNA-binding, excluding hotspot (%)	Others (%)
Colorectal (n=49)	16 (33)	6 (12)	11 (22)	10 (20)	7 (14)	24 (49)	20 (41)	5 (10)
Ovarian (n=31)	9 (29)	4 (13)	4 (13)	10 (32)	4 (13)	7 (23)	21 (68)	3 (10)
GI, other (n=29)	9 (31)	2 (7)	10 (35)	7 (24)	4 (14)	8 (28)	22 (76)	2 (7)
Lung (n=18)	6 (33)	6 (33)	2 (11)	2 (11)	2 (11)	3 (17)	13 (72)	2 (11)
Genitourinary (n=17)	3 (18)	3 (18)	4 (23)	4 (23)	4 (23)	4 (24)	10 (59)	4 (24)
GYN, other (n=18)	2 (11)	4 (22)	3 (17)	5 (28)	4 (22)	6 (33)	9 (50)	3 (16)
Head and neck (n=15)	3 (20)	4 (26)	4 (26)	2 (13)	3 (20)	3 (20)	12 (80)	2 (13)
Breast (n=22)	7 (32)	2 (9)	3 (14)	5 (23)	6 (27)	6 (27)	14 (64)	5 (23)
Sarcoma (n=9)	3 (33)	1 (11)	0	3 (33)	2 (22)	1 (11)	6 (66)	2 (22)
Endometrial (n=9)	3 (33)	1 (11)	4 (44)	0	1 (11)	3 (33)	5 (56)	1 (11)
Melanoma (n=8)	2 (25)	4 (50)	1 (12)	0	2 (25)	2 (25)	6 (75)	2 (25)
Pancreatic (n=5)	0	1 (20)	3 (60)	1 (20)	0	2 (40)	3 (60)	0
Thyroid (n=4)	0	0	1 (25)	2 (50)	1 (25)	1(25)	2 (50)	1 (25)
Other (n=39)	10 (26)	7 (18)	13 (33)	8 (20)	7 (18)	10 (26)	26 (67)	7 (18)
Total (n=273)	73 (27)	45 (16)	63 (23)	59 (22)	47 (17)	80 (29)	169 (62)	39 (14)

* Tumors can have more than 1 mutation in different exon and codon locations. Abbreviations: GI-gastrointestinal, GYN-gynecological.

When all screened patients were considered, p53 mutations in the hot-spot codons (175, 245, 248, 249, 273, and 282) were significantly more common in colorectal cancer (19/101, 19%) than in the other tumor types (33/580, 6%). When the analysis was limited to patients with p53 mutations, 24 (49%) patients with colorectal cancer had hot-spot mutations compared to 60 (27%) patients with the remaining tumor types (p=.0002). Mutations in the DNA-binding domain excluding the hot-spot codons were more commonly seen in ovarian and lung cancer (Supplemental-Figure 1a-1g).

### Other Aberrations

We examined aberrations other than p53 in patients with p53 mutations and wild-type p53. Additional aberrations were found more often in the group with p53 mutations than in the wild-type p53 group (p=.002). The distribution of the number of other aberrations in the two groups is summarized in Table 3. Interestingly, PTEN loss or mutation was found more often in tumors with p53 mutations than in those with wild-type p53 (23% vs. 5%, p=.0005). Similarly, HER2 aberrations were significantly more common in tumors with p53 mutations than in those with wild-type p53 (8% vs. 2%, p=.003). Alternatively, there was a trend toward a higher percentage of *BRAF* mutations in the patients with wild-type p53 compared with those with mutated p53 (6% vs. 2%, p=. 07).

**Table 3:** Number of other aberrations associated with wild-type and mutant p53

No. of other aberrations	P53 mutation N=273 (%)	P53 wild-type N=418 (%)	P-value
0	88 (32.2)	162 (38.8)	.0018
1	79 (28.9)	134 (32.1)	
2	38 (13.9)	68 (16.3)	
3	19 (7.0)	29 (6.9)	
≥4	49 (18)	25 (6)	

The distribution of the common additional aberrations in the groups of patients with mutant and wild-type p53 is summarized in Table 4. The most common additional aberrations seen in the mutant p53 group were PTEN (23%), *KRAS* (16%), *APC* (12%), and *PI3K* (10%). The most common aberrations seen in the wild-type p53 group were *KRAS* (16%), *PI3K* (14%), and PTEN (9%). Overall, aberrations involved in the RAS/RAF/MEK pathway were more frequent in the p53 mutant group than in the wild-type p53 group (59/142 [42%] vs. 81/258 [31%], respectively; p=.04). In contrast, no difference was observed between the two groups in aberrations involving the PI3K/AKT/mTOR pathway (56/142 [39%] vs. 88/258 [34%], respectively; p=.29).

**Table 4:** Distribution of the most common other mutations associated with both wild-type and mutant p53 status

	P53 mutation (N=273)			P53 wild-type (N=418)			P
	Tested	Positive	%	Tested	Positive	%	
PTEN*	93	21	23	336	31	9	.0005
PIK3CA	263	26	10	388	55	14	.10
BRAF	250	6	2	329	18	6	.07
KRAS§	255	40	16	363	57	16	1.0
EGFR§	241	9	4	334	7	2	.24
c-Met	254	9	4	370	11	3	.69
HER2§	209	16	8	313	7	2	.003
APC	119	14	12	163	12	7	.21

* : PTEN loss by immunohistochemistry or mutation

§: Mutation or amplification.

### Treatment

Of the 273 patients with p53 mutations, 195 (71%) were treated on various phase I clinical trials; among them, 20 (7%) patients received local therapy, including various hepatic arterial infusion and intra-peritoneal therapy regimens. The remaining 78 (29%) patients did not receive any treatment for various reasons. Of 418 patients with wild-type p53, 312 (75%) were treated on various phase I clinical trials; among them, 26 (6%) patients received local therapy. The remaining 106 (25%) patients did not receive any therapy. The reasons for which patients did not receive treatment were ineligibility or unavailability of a clinical trial (n=44, 24%), insurance issues (n=9, 5%), and personal reasons (n= 131, 71%).

Among patients with additional aberrations in the p53 mutation group, 37 (26%) patients received targeted therapy against the additional aberrations, 62 (43%) patients received non-matched therapy, and 44 (31%) patients received no therapy. In the wild-type p53 group, 78 (30%) patients received targeted therapy against the additional aberrations, 117 (45%) patients received non-matched therapy, and 63 (25%) patients received no therapy.

### Overall Survival

There was no difference in overall survival between patients with p53 mutations and those with wild-type p53 (median survival, 14 months, 95% confidence interval: 11-17, for both groups; hazard ratio = 1.0, 95% confidence interval: 0.8-1.3; p=.71). The overall survival of treated and untreated patients with p53 mutations is shown in Figure 1a. The median survival durations were 14.6 months and 7.2 months, respectively (p=.006). The overall survival of treated and untreated patients with wild-type p53 is shown in Figure 1b. The median survival durations were 15.8 months and 9.3 months, respectively (p=.0002).

**Figure 2:**
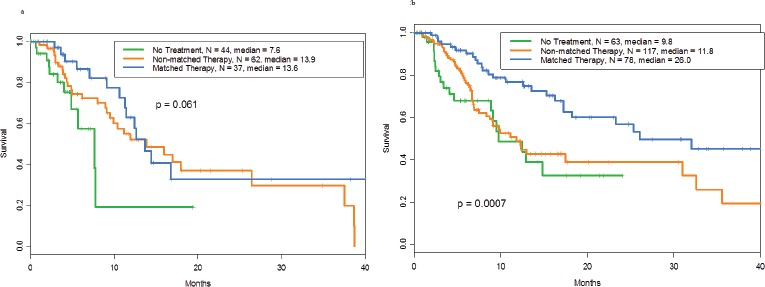
**a** Overall survival of patients with mutant p53 tumors by whether they received matched targeted therapy (n=37)-, non-matched (n=62)-, or no therapy (n=44) for the other aberrations with a median survival of 13.6, 13.9 and 7.6 months, respectively. **b.**Overall survival of patients with wild-type p53 tumors by whether they received matched targeted therapy (n=78)-, non-matched (n=117)-, or no therapy (n=63) for the other aberrations with a median survival of 26, 11.8 and 9.8 months, respectively.

The overall survival of patients with p53 mutations by exon is shown in Supplemental-Figure 2. The median overall survival durations for exon 5, 6, 7, and 8 mutations were 9.5, 13.6, 15.7, and 17.6 months, respectively (p=.31).

### Overall Survival by p53 Status and Additional Aberrations

We performed survival analyses of patients with p53 mutations and additional aberrations. The median survival durations were 13.6 months in patients who received matched therapy (n= 37), 13.9 months in those who received non-matched therapy (n= 62), and 7.6 months in those who received no treatment (n= 44) (p= .06; Figure 2a). In multivariate analyses (in which performance status [0 vs. ≥^1^], serum albumin level [<^3^.^5^ g/dL vs. ≥^3^.^5^ g/dL], lactate dehydrogenase level [<617 U/L vs. ≥617 U/L], number of metastatic sites [0-2 vs. ≥^3^], liver metastases, and number of prior treatments [0-3 vs. ≥^4^] were taken into consideration), no significant difference in survival was noted among the three groups (p = 0.15).

In patients with wild-type p53 and other aberrations, those treated with matched therapy (n= 78) had longer survival than those treated with non-matched therapy (n= 117) or those who received no treatment (n= 63) (median survival, 26.0 vs. 11.8 vs. 9.8 months, respectively; p= .0007; Figure 2b).

A multivariate analysis (after adjustment for the variables listed above) confirmed this difference between the treatment groups (p=.0002). These findings translated into longer survival for the patients with a more number of additional mutations in the wild-type p53, but not the mutant p53 group (Supplemental-Figure 3a and 3b).

## DISCUSSION

This analysis expands on our previous report on the characteristics and survival of patients with advanced cancer and p53 mutations. In the current study, we evaluated the effects of specific p53 mutations and additional aberrations on survival in patients referred to the Phase I Clinical Trials Program. P53 mutations were associated with higher numbers of other aberrations and resistance to targeted therapy. P53 status did not affect overall survival in this patient population, but matched therapy for other aberrations was associated with longer survival in patients with wild-type p53. P53 mutations varied by tumor type and were associated with higher proportions of PTEN and HER2 aberrations than wild-type p53.

Overall, 39.5% of the patients analyzed had a p53 mutation. Patients with p53 mutations were older (p<.0001) and had higher rates of hepatic metastases (p=.005) than patients with wild-type p53. P53 mutations within exon 5 were more common in colorectal cancer, while mutations within exons 6, 7, and 8 were more common in lung, pancreatic, and ovarian cancer, respectively.

Our findings of older age, more frequent liver metastases, and more frequent PTEN loss in the p53-mutated group in the current, more extensive analysis, were similar to those in our prior analysis [15].

Our results are consistent with previously known data showing that the percentage of p53 mutation varies by tumor type and ranges from 10% to 80% [16]. Our results are also in line with a previous study showing that p53 mutations occurred more frequently in older patients with rectal cancer [17]. Other investigators have reported that breast cancers harboring p53 mutations occur more often in young women (age ≤40 years at diagnosis)[18]. In the latter study p53 mutations were found in 63.8% of patients, and the percentage of patients with triple negative breast cancer was not reported.

The observation that patients with p53 mutations had more liver metastases may be partially explained by the role of mutant p53 in promoting cell migration and invasion [19], as shown in preclinical models [11, 20]. A previous study showed that patients with colorectal cancer with p53 mutations had a larger number of hepatic metastases than patients with wild-type p53 tumors [21].

The distribution of various types of p53 mutations in our study varies by tumor type. This difference may contribute to the variable prognostic value of p53 mutations in various tumor types with p53 mutations [13].

In our series, tumors with p53 mutations had more additional aberrations than tumors with wild-type p53 (p=.002) (Table 3). We found that loss of PTEN or PTEN mutation (23% vs. 5%, p=.0005) and HER2 aberrations (8% vs. 2%, p=.003) were more common in tumors with p53 mutations than in those with wild-type p53. These results may be due in part to the role of p53 in maintaining genomic integrity in mammalian cells, as previously described [22]. The frequency of PTEN aberrations in tumors with p53 mutations further supports the need for “individualized” treatment targeting the PI3K/AKT/PTEN pathway in prospective clinical trials.

No difference in survival was observed between patients with p53 mutations and those with wild-type p53 (p=0.71). In both groups, the overall survival duration was longer in patients who received treatment than in those who did not. However, among patients with wild-type p53 and other aberrations, survival was superior in those who received matched therapy against the additional aberrations compared to non-matched therapy. We have previously published evidence that identifying specific molecular abnormalities and choosing therapy based on these abnormalities is associated with a longer time to treatment failure in the phase I setting than that of previous systemic therapy. Furthermore, in the non-randomized setting, rates of response, time to treatment failure, and survival were higher with matched targeted therapy than with non-matched therapy [23].

An intriguing finding in the current report is the observation that patients with wild-type p53 and other aberrations who received matched therapy against these aberrations had longer survival than those treated with non-matched therapy or those who received no treatment (median survival, 26 vs. 11.8 vs. 9.8 months, respectively; p=.0007). This survival advantage was confirmed in multivariate analysis after adjustment for other covariates (p=.0002). In contrast, in the p53 mutation group with additional aberrations, the survival durations were similar for those who received matched therapy against the additional aberrations, those treated with non-matched therapy, and those who received no therapy (median survival, 13.6 vs. 13.9 vs. 7.6 months, respectively; p=.06).

The exact role of p53 in the context of other molecular aberrations and their prognostic significance in various tumor types needs to be elucidated. Whether p53 mutations are driver mutations or contribute to the emergence of resistance to targeted agents needs to be evaluated. Resistance to therapy seen in tumors with p53 mutation requires the development of new agents or strategies. Several investigators have reported that p53-based cyclotherapy may represent a successful strategy. [24-30] This approach is based on protection of normal cells from chemotherapy-induced adverse events. Low-doses of p53 activators, such as nutlin-3 and actinomycin D, are used to induce p53-dependent cell cycle arrest in normal cells bearing wild-type p53. [24-30] Prospective clinical trials are needed to explore the role of this approach in overcoming p53 resistance.

Our data suggest that the identification of driver mutations in patients with wild-type p53 and other aberrations and the selection of targeted therapy may contribute to improved survival. Recently published data in head and neck squamous cell carcinoma also demonstrated that PI3K/Akt/mTOR inhibition using PF-04691502 is enhanced with induction of wild-type p53 in human xenograft and murine knockout models [31]. The authors concluded that p53 is one of the potential modifiers of response (in addition to PI3KCA, PTEN, TGF-β alterations) [31].

In conclusion, our data add to the published data demonstrating that p53 mutations are associated with a poor prognosis and resistance to treatment, and they emphasize the need to develop agents or strategies to overcome this resistance. These findings further support the need to individualize cancer therapy and should be validated in carefully designed prospective trials.

## METHODS

### Patients

We reviewed all patients who underwent testing for p53 mutation status in the Department of Investigational Cancer Therapeutics at MD Anderson Cancer Center. Molecular profiling was performed on available tissue samples from consecutive patients with advanced tumors referred to the Clinical Center for Targeted Therapy. Patients were of various ages and had advanced or metastatic cancer that was refractory to standard therapy, that had relapsed after standard therapy, or for which there was no standard therapy available. All protocols required that participants have evidence of evaluable or measurable disease according to Response Evaluation Criteria in Solid Tumors (RECIST) guidelines [32, 33] and an Eastern Cooperative Oncology Group performance status of 0-2. Additional eligibility criteria varied by protocol. All patients provided written informed consent prior to enrollment in a trial. All trials, as well as this analysis, were performed with the approval of and in accordance with the guidelines of the MD Anderson Institutional Review Board.

### Analysis of Molecular Aberrations

The p53 mutation status was determined by either polymerase chain reaction–based or next-generation sequencing in a Clinical Laboratory Improvement Amendments–certified laboratory, as previously described [23]. The next-generation sequencing included 182 genes in targeted next-generation sequencing Foundation One platform (Foundation Medicine, Cambridge, MA).

### Therapy

Treatment was selected as previously published [23]. Briefly, the allocation of patients to investigational treatment varied over time according to protocol availability, eligibility criteria, histologic diagnosis, the patient's prior response to therapy, potential toxicity, insurance coverage, and patient preference or physician choice. The assignment to a clinical trial was determined after clinical, laboratory, and pathologic data from all available patient records were reviewed. Patients whose tumors had a molecular aberration were treated on a clinical trial with a matched targeted agent, when available.

### Endpoints and Statistical Methods

Patients' characteristics were analyzed using descriptive statistics. Categorical data were described using contingency tables, including counts and percentages. Continuously scaled measures were summarized by median and range. The association between two categorical variables was examined using the chi-square test. Survival and hazard functions were estimated using the Kaplan-Meier method, and survival between groups was compared using the two-sided log-rank test. Survival was analyzed according to the type of p53 mutations and the treatment. Hazard ratios with and without adjustment for potential confounding variables were estimated using Cox proportional hazard regression analysis.

## SUPPLEMENTARY AND FIGURES


