# Supplementary material for: Characteristics and survival of patients with advanced cancer and p53 mutations
Source: Oncotarget. 2014 May 25;5(11):3871–9. doi: 10.18632/oncotarget.2004 (PMC4116527; doi:10.18632/oncotarget.2004)
Supplement: Supplementary file 1 [file oncotarget-05-3871-s001.pdf]

Characteristics and survival of patients with advanced cancer and p53 mutations

Supplementary Material

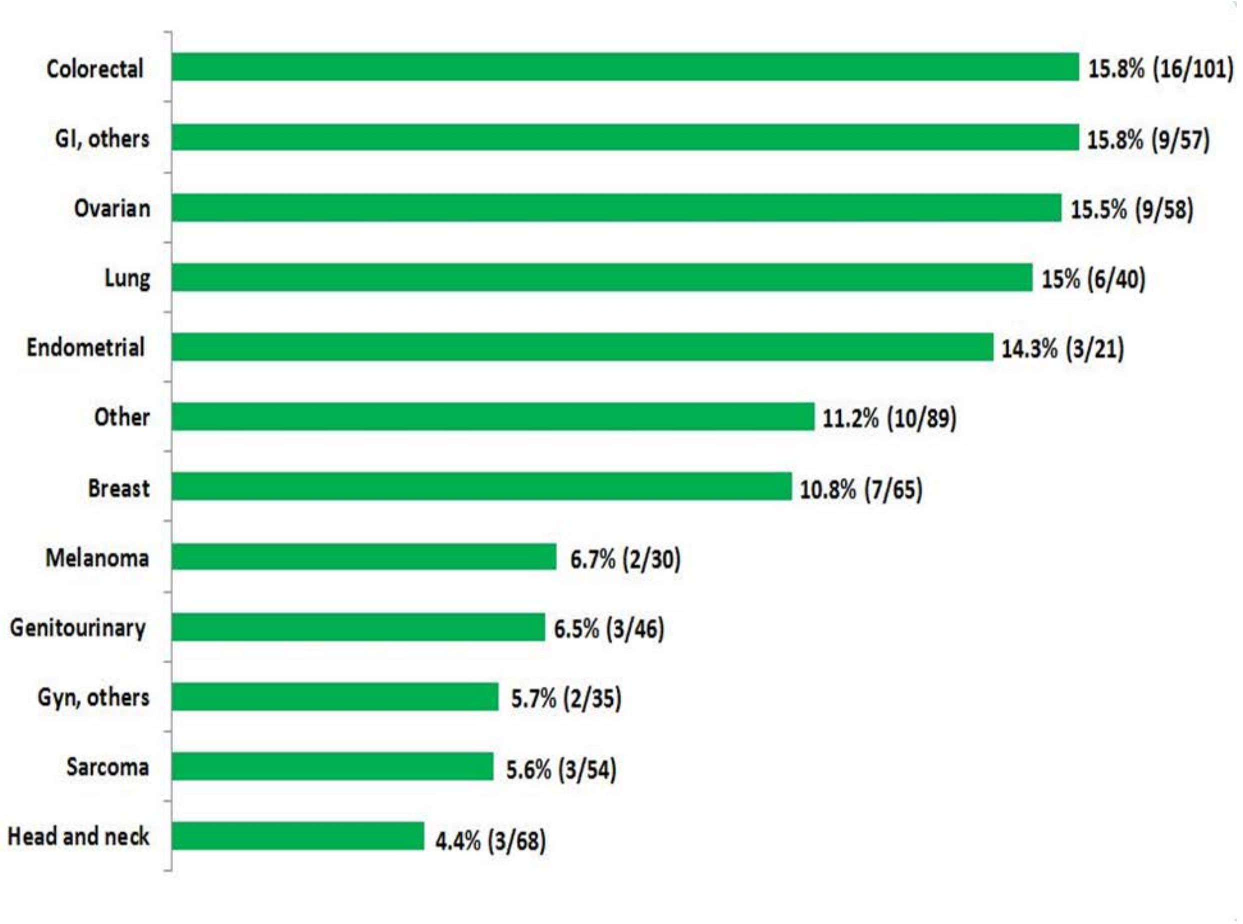

Supplemental Figure 1a: Prevalence of various p53 mutations within Exon 5

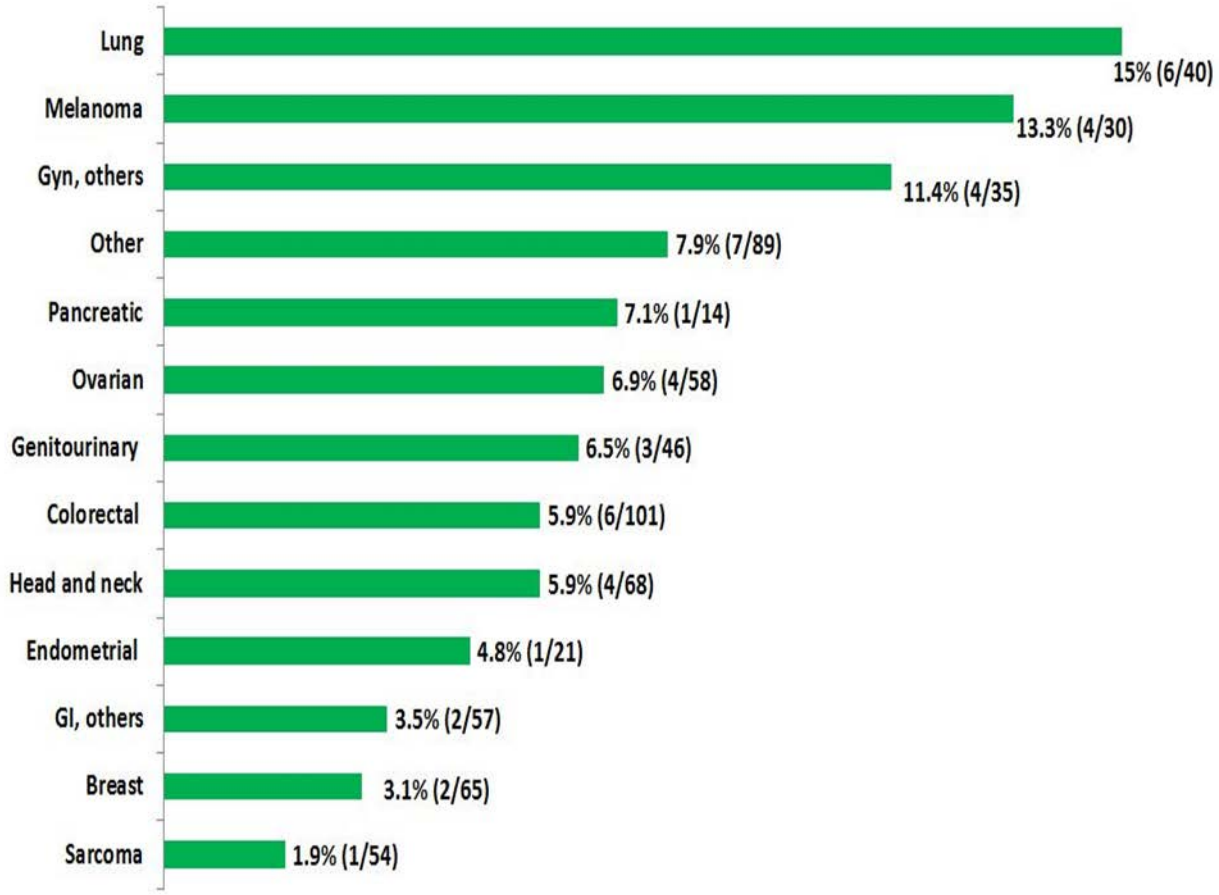

Supplemental Figure 1b: Prevalence of various p53 mutations within Exon 6

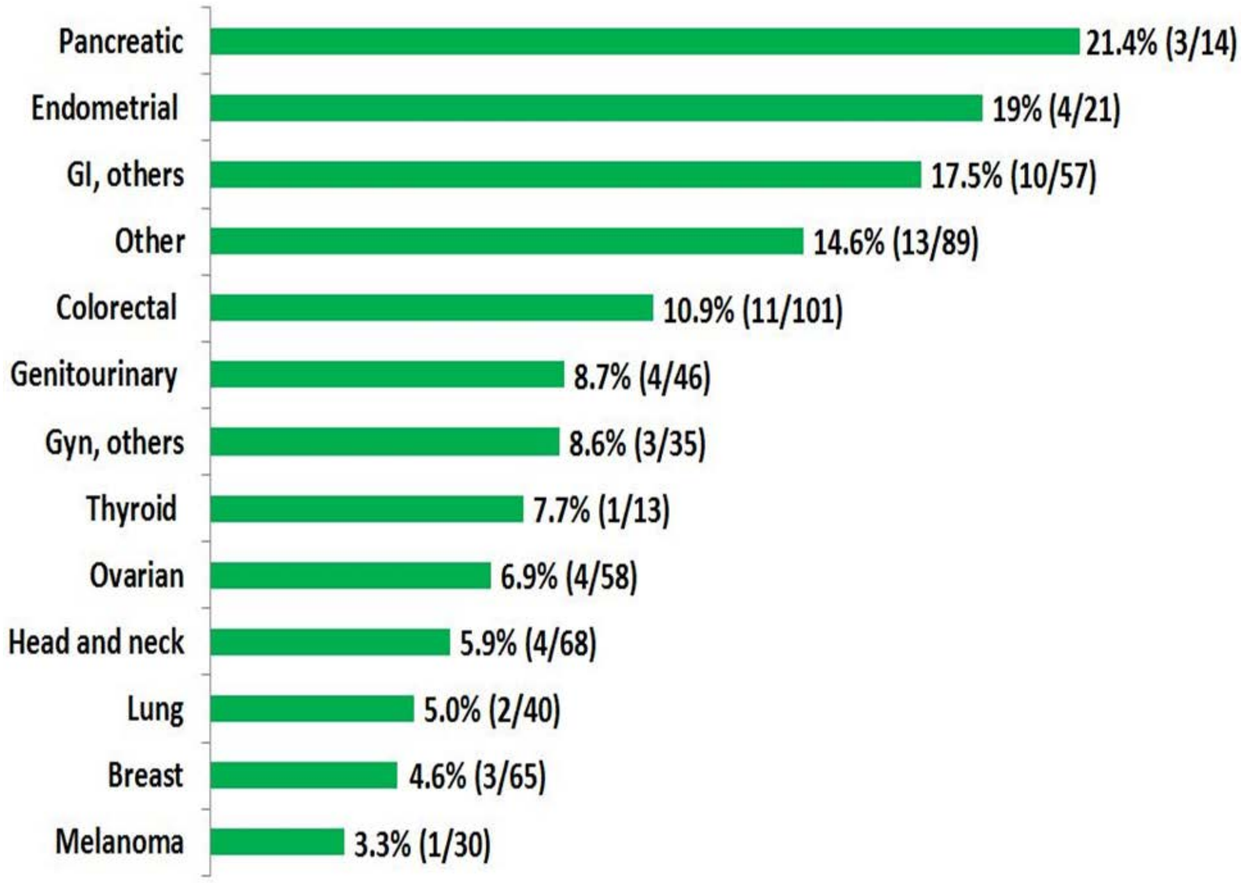

Supplemental Figure 1c: Prevalence of various p53 mutations within Exon 7

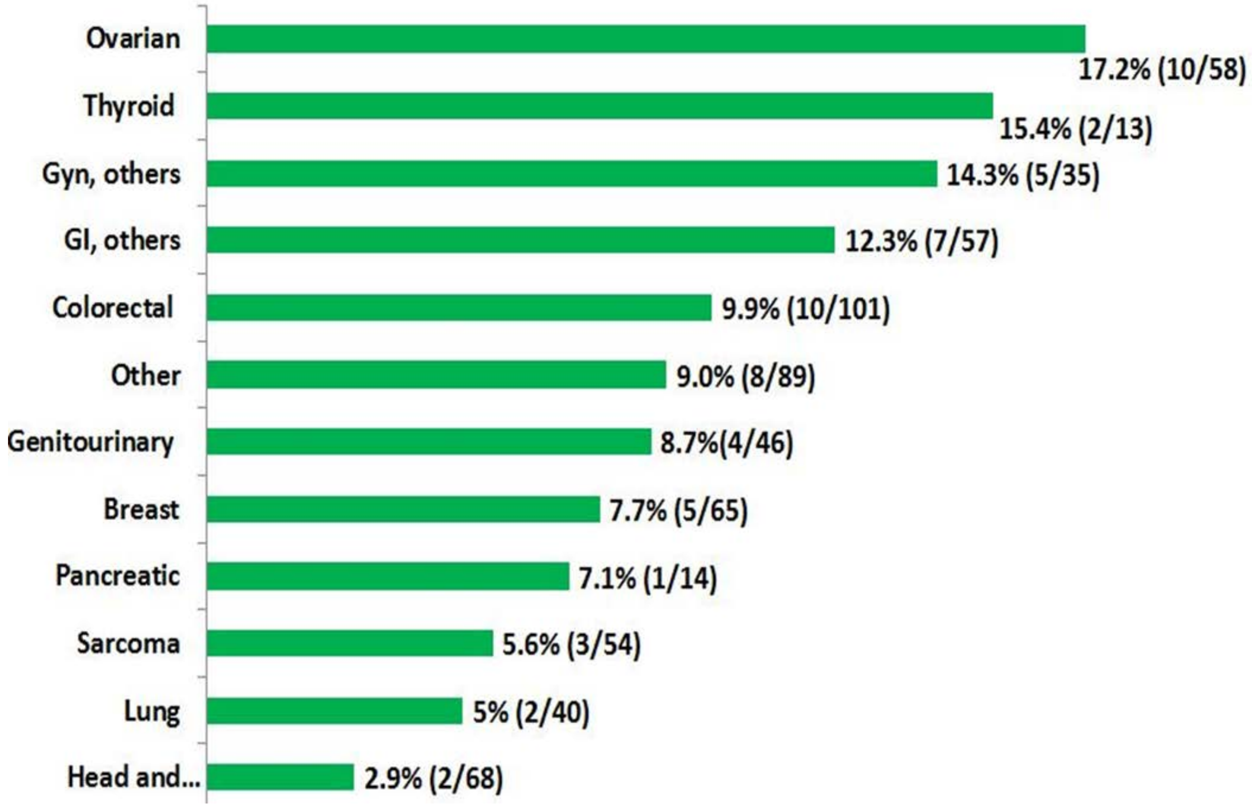

Supplemental Figure 1d: Prevalence of various p53 mutations within Exon 8

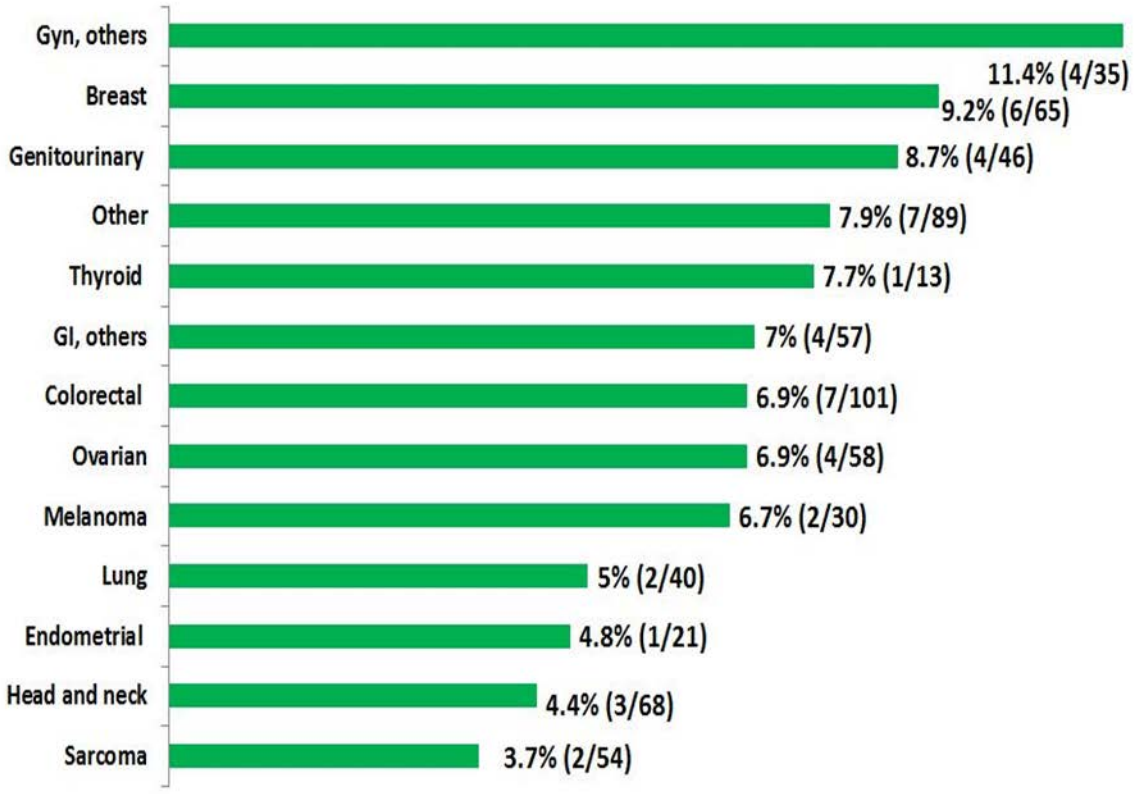

Supplemental Figure 1e: Prevalence of various p53 mutations within other Exon

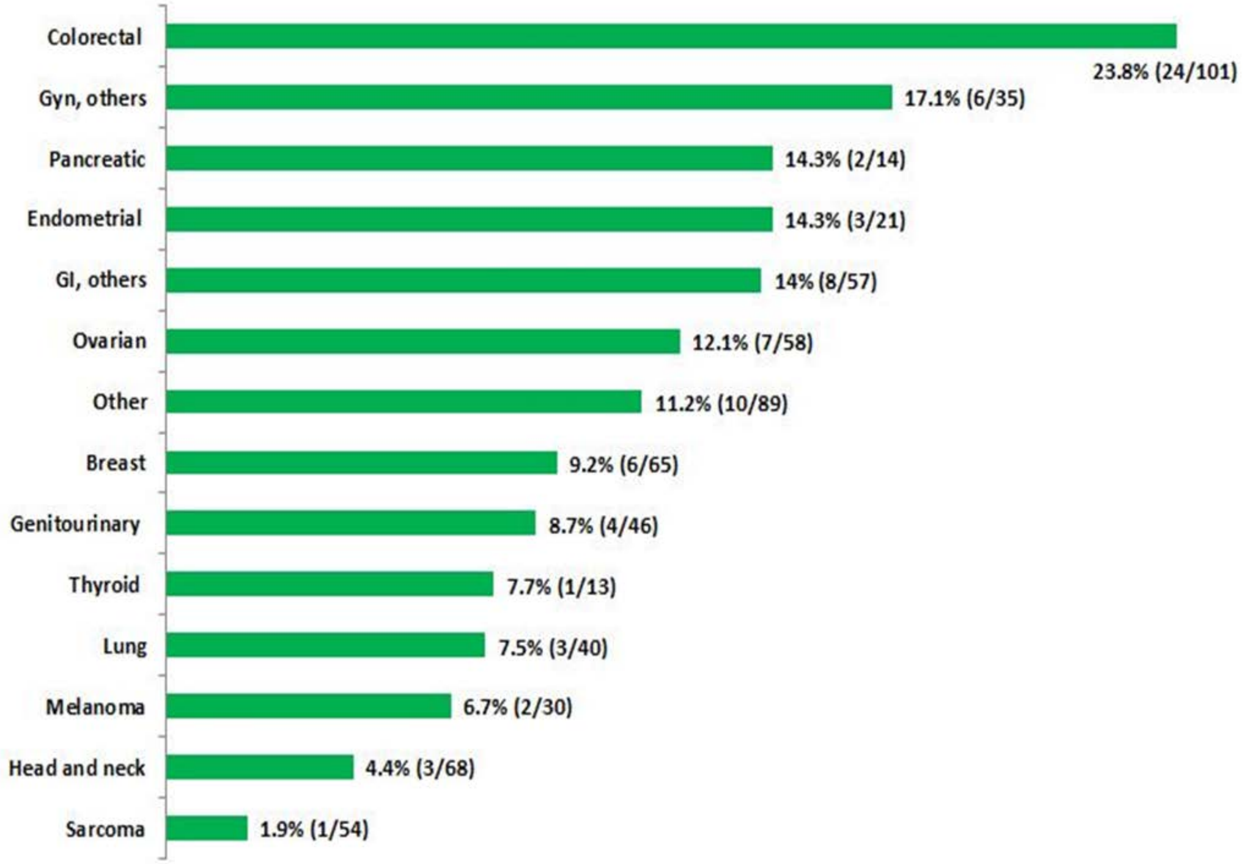

Supplemental Figure 1f: Prevalence of various p53 mutations within Hot-spot Codons

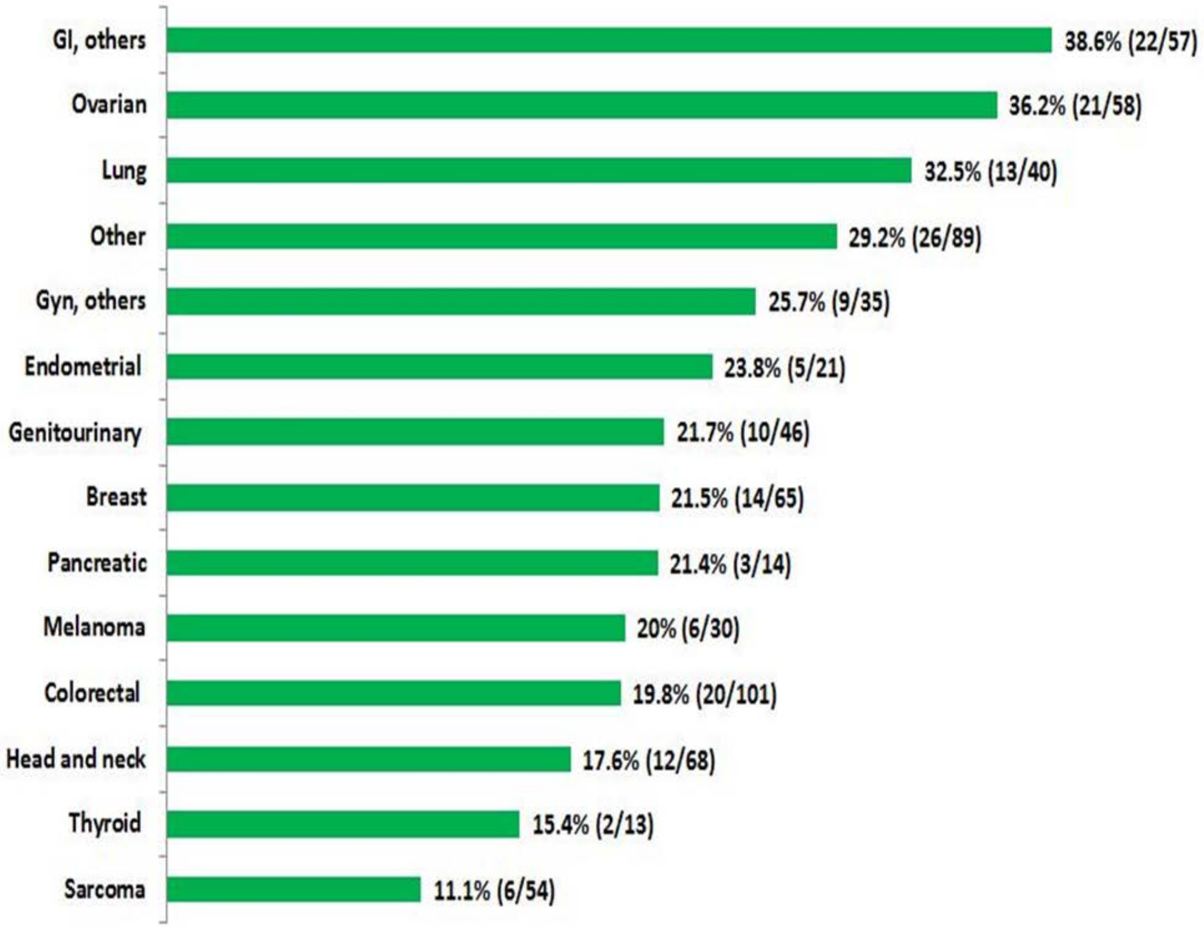

Supplemental Figure 1g: Prevalence of various p53 mutations within DNA-binding domain, excluding hot-spot codons

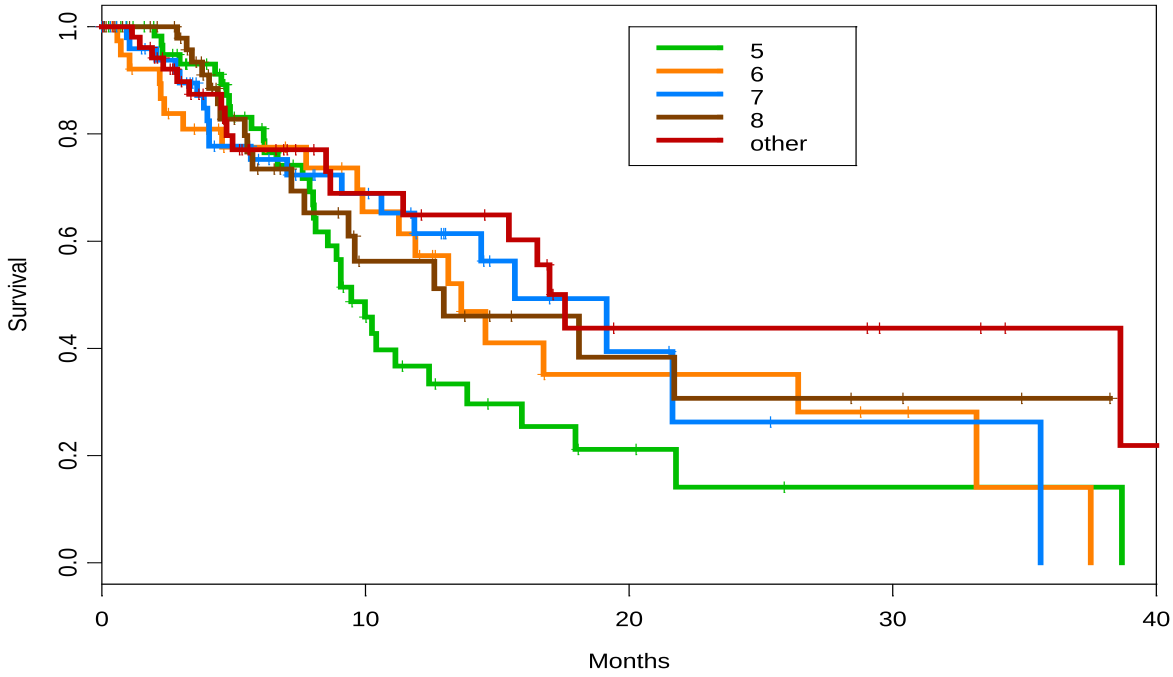

Supplemental Figure 2. Overall survival by various exons

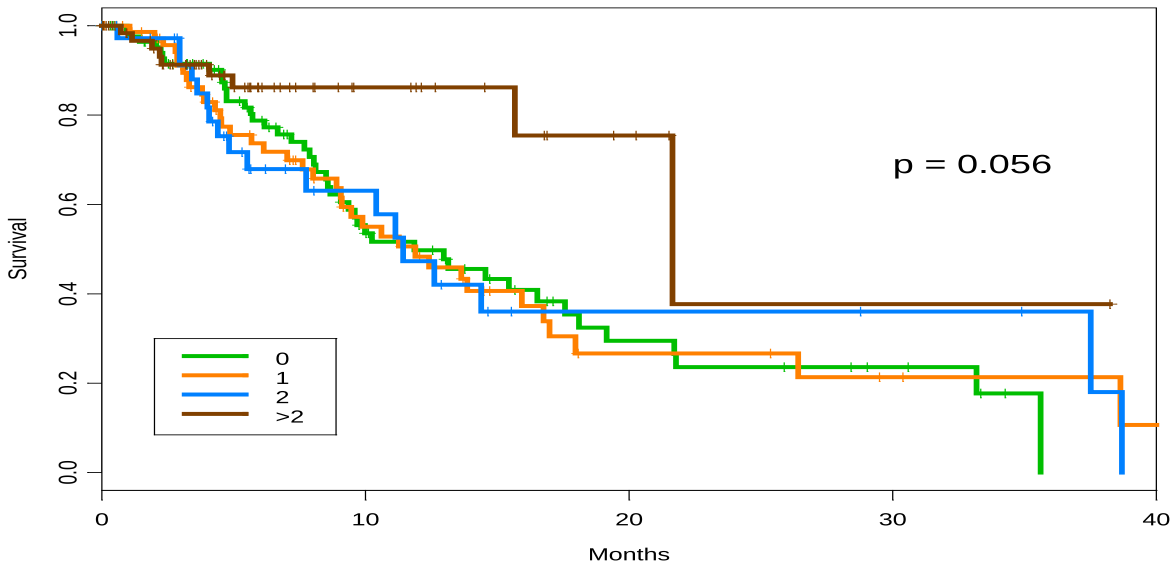

Supplemental Figure 3a: Overall survival categorized by the number of other aberrations in mutate p53d

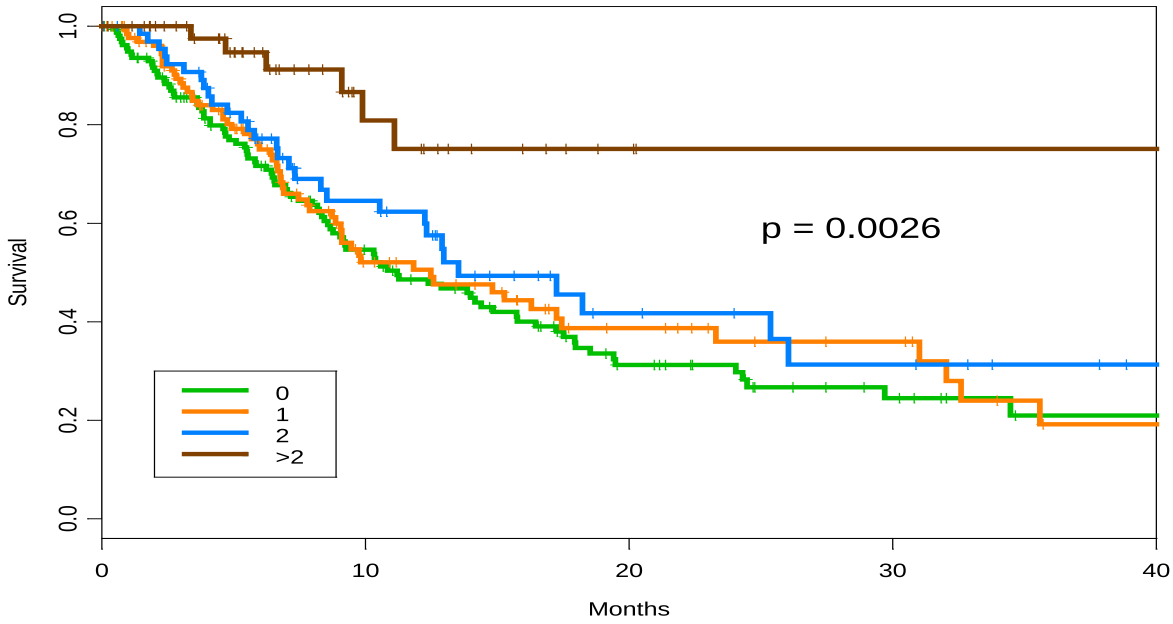

Supplemental Figure 3b: Overall survival categorized by the number of other aberrations in wild-type p53
